# A synthetic ‘*essentialome*’ for axenic culturing of ‘*Candidatus* Liberibacter asiaticus’

**DOI:** 10.1186/s13104-022-05986-5

**Published:** 2022-04-01

**Authors:** Lulu Cai, Mukesh Jain, Alejandra Munoz-Bodnar, Jose C. Huguet-Tapia, Dean W. Gabriel

**Affiliations:** Department of Plant Pathology, University of Florida, Gainesville, FL 32611 USA

**Keywords:** ‘*Candidatus* Liberibacter’ spp., Citrus greening, Conjugation, Huanglongbing, *Liberibacter crescens*, Minimal genome, Essentialome

## Abstract

**Objective:**

*‘Candidatus* Liberibacter asiaticus’ (CLas) is associated with the devastating citrus ‘greening’ disease. All attempts to achieve axenic growth and complete Koch’s postulates with CLas have failed to date, at best yielding complex cocultures with very low CLas titers detectable only by PCR. Reductive genome evolution has rendered all pathogenic ‘*Ca.* Liberibacter’ spp. deficient in multiple key biosynthetic, metabolic and structural pathways that are highly unlikely to be rescued in vitro by media supplementation alone. By contrast, *Liberibacter crescens* (Lcr) is axenically cultured and its genome is both syntenic and highly similar to CLas. Our objective is to achieve replicative axenic growth of CLas via addition of missing culturability-related Lcr genes.

**Results:**

Bioinformatic analyses identified 405 unique ORFs in Lcr but missing (or truncated) in all 24 sequenced CLas strains. Site-directed mutagenesis confirmed and extended published EZ-Tn*5* mutagenesis data, allowing elimination of 310 of these 405 genes as nonessential, leaving 95 experimentally validated Lcr genes as essential for CLas growth in axenic culture. Experimental conditions for conjugation of large GFP-expressing plasmids from *Escherichia coli* to Lcr were successfully established for the first time, providing a practical method for transfer of large groups of ‘*essential*’ Lcr genes to CLas.

**Supplementary Information:**

The online version contains supplementary material available at 10.1186/s13104-022-05986-5.

## Introduction

*‘Candidatus* Liberibacter’ spp. are a versatile group of fastidious, Gram-negative, psyllid-transmitted and phloem-limited *α*-Proteobacteria (order *Rhizobiales*). ‘*Ca.* Liberibacter’ spp. have a wide host range and are associated with several plant diseases of variable economic consequence, some high enough to warrant regulatory action. Huanglongbing (HLB) or citrus ‘greening’ is associated with ‘*Ca.* L. asiaticus’ and ‘*Ca.* L. americanus’ (CLas and CLam, both vectored by Asian citrus psyllid *Diaphorina citri*) and ‘*Ca.* L. africanus’ (vectored by African citrus psyllid *Trioza erytreae*) [1]. Aberrant assimilate partitioning and nutrient transport leads to progressive decline in productivity and eventual death of the HLB-infected trees. *Liberibacter crescens* (Lcr) strain BT-1 (NC_019907.1) was originally isolated from leaf sap of a diseased Babaco Mountain papaya (*Carica stipulata*×*C. pubescens*) and has been axenically cultured in vitro [2]. Lcr BT-1 has no known plant or insect host and serves as a surrogate gene expression host and model for functional genomics of CLas [3]. Comparative metagenomic analyses suggested stepwise reductive evolution of all ‘*Ca*. Liberibacter’ spp. including 24 fully sequenced CLas strains (all genomes ∼ 1.2 Mb) from a common ancestor following an initial split of Lcr (1.5 Mb genome) from other *Rhizobiales* [4]. All attempts to fulfill Koch’s postulates or to culture CLas in axenic media have failed. Only inconsistent, transient and very low “titer” co-cultures [5–9] have been obtained, rendering their use impractical for most functional genomics purposes designed to understand host/pathogen/vector interactions and implement effective disease mitigation strategies.

## Main text

### HLB pathosystem (CLas/citrus host/psyllid vector) exists as a ‘holobiont’

CLas survival and slight growth within complex host-derived microbial communities was detected by PCR in cocultures of Ishi-1 [6] and psy62 [7, 8]. CLas strain A4 titers increased modestly in leaf disc explants incubated in the presence of glucose and the antibiotic amikacin under microaerobic conditions [10]. Li et al. [11] reported a 419-fold increase of CLas density without any corresponding increase in other citrus phloem-associated microflora in dodder (*Cuscuta campestris*) tendrils trained on CLas-infected citrus.

These observations indicate that the HLB pathosystem (CLas/citrus host/psyllid vector) exists as a ‘holobiont’ (host/vector with its endo- and extracellular microbiome) [12]. Metabolic and ecological interactions (mutualistic, synergistic or competitive) between the microbial community members within the HLB pathosystem are paramount for the survival of CLas with its highly reduced genome [4]. Genome reduction is a dominant mode of evolution in intracellular pathogenic/endosymbiotic bacteria, providing robust niche-specificity by virtue of increased metabolic efficiency and decreased transcriptional and regulatory costs associated with a streamlined genome [13–15].

### Gene ‘*essentiality*’ is non-binary and context-specific

Peterson and Fraser [16] have argued against a universal, theoretically rigid ‘minimal genome’ or ‘*essentialome*’ design for achieving an autonomous self-replicating cellular unit based on gene conservation criteria across large phylogenetic distances. Mounting evidence suggests that the gene ‘*essentiality*’ concept is neither binary nor static and evolves [17, 18] under specific environmental and contextual genomic constraints [19, 20]. For instance, approximately one-third of the essential genes in *E. coli* are non-essential in *Bacillus subtilis* and vice versa [21]. Likewise, ~ 17% of genes considered essential in the budding yeast *Saccharomyces cerevisiae* are non-essential in the fission yeast *Schizosaccharomyces pombe* and ~ 27% of essential fission yeast genes are non-essential in the budding yeast [22]. Some ‘*essential*’ genes are dispensable in the context of another missing gene (or pathway) because the former might encode protective functions towards the (likely) toxic effects of the latter. For example, glyoxalase I (GloA) is a biologically fundamental and ubiquitously conserved enzyme for detoxification of methylglyoxal, a cytotoxic byproduct of glycolysis. However, absence of *gloA* is well tolerated in CLas because of transcriptional downregulation of glycolysis and subsequent reliance on scavenging ATP from host cells by virtue of an *nttA*-encoded ATP/ADP translocase present in the uncultured pathogenic ‘*Ca.* Liberibacter’ spp. [23].

### Successful axenic culturing of CLas requires a synthetic ‘*essentialome*’

Genomic and metabolic pathway comparisons between Lcr and all pathogenic ‘*Ca.* Liberibacter’ spp. revealed a trend for the reduction or complete absence of multiple biosynthetic pathways, metabolic enzymes and secretion systems consistent with their intracellular lifestyle [4]. Even though the genomes of Lcr and pathogenic ‘*Ca.* Liberibacter’ spp. are highly similar and microsyntenic [2, 4], the core Liberibacter genomes share only 658 genes, and most of the species-specific genes encode hypothetical proteins. Bioinformatic analysis revealed that 37% of the functionally annotated genes in the Lcr genome are species-specific in comparison to only 17% in CLas and 9% in CLam [24].

**Fig. 1:**
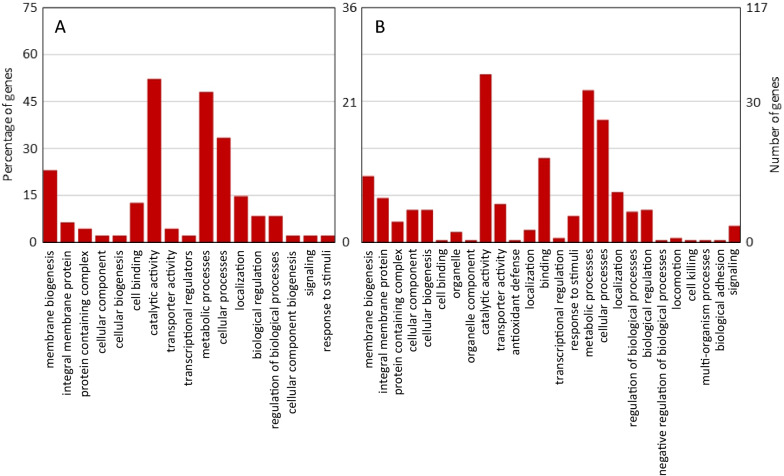
Metabolic/physiological and structural differences between cultured, non-pathogenic *Liberibacter crescens* and uncultured, pathogenic ‘*Ca.* L. asiaticus’. Clusters of Orthologous Genes (COG) analysis reveals functions encoded by 302 *L. crescens* genes that are either (**A**) completely absent (81 genes) or (**B**) truncated (221 genes) in all ‘*Ca.* L. asiaticus’ genomes sequenced to date

Based on genome scale metabolic modeling and large-scale gene ‘*essentiality*’ data sets across 79 bacterial and archaeal domains, the ‘*essentiality*’ patterns cluster together phylogenetically in silico as well as experimentally at the metabolic pathway level [25]. It is therefore axiomatic that the genes validated as ‘*essential*’ for Lcr growth in vitro [23, 26–29] but absent in CLas, are likely indispensable for maintaining CLas in replicative cultures. Under this premise, unique gene loci in Lcr were identified using a custom Perl script and reciprocal blasts implemented in the OrthoMCL software [30] at e-value cut offs of < 3e–30 and < 40% identity. Functional annotation by InterProScan [31] and Prokka [32] revealed 405 unique ORFs (including 104 hypothetical proteins) in Lcr that were missing in all sequenced strains of pathogenic Liberibacters (Additional File 1). Out of these, 120 ORFs (81 annotated and 39 hypothetical proteins) were completely absent (Additional File 2) whereas 286 ORFs (221 annotated and 65 hypothetical proteins) were truncated in the genomes of all sequenced CLas strains. Classification of the 302 annotated ‘*essential*’ genes of Lcr into Cluster of Orthologous Groups (COG) is presented in Fig. 1. Notably, a relatively high percentage of these were involved in membrane or envelope biogenesis and partitioning. Tan et al. [33] very recently reported a similar number (323) of COGs unique to Lcr using very different methodology.

The ‘*essentiality*’ of 405 genes for Lcr growth was validated by targeted site-directed marker interruption [3] and compared with previously published EZ-Tn*5* transposon mutagenesis dataset [27]. EZ-Tn*5* mutagenesis data were also manually examined to determine if the location of the Tn*5* insertions within each ORF likely affected the expression of the predicted conserved domains in the ORF, either by polar effects or by direct disruption. Some Tn*5* insertions occurring in the terminal 20% of the 3ʹ region of the target ORF and outside of known functional protein domains were considered not likely to produce a mutated phenotype [34] and were experimentally validated by multiple failed knockout attempts, as were observed for Lcr glyoxalase (*gloA*) [23] and Kdo2-lipid IVA lauroyltransferase (*lpxXL*) [28, 29] genes. Out of the 405 unique Lcr genes, 310 were eliminated in this study as being likely non-essential, leaving 95 genes (65 annotated and 30 hypothetical proteins) that were either ‘*essential*’ or quasi-essential for Lcr growth (Additional File 3).

### Nutrient reprieve alone is likely insufficient for axenic growth of CLas

Large-scale computational metabolic modeling identified 372 genes driving 892 metabolic reactions involving 887 metabolites in Lcr BT-1. By comparison, only 253–285 genes, driving 814–840 reactions and producing 802–837 metabolites were identified in six different CLas strains [35]. In addition to 109 unique metabolic reactions present in Lcr, ~ 30% of the Lcr-specific reactions were associated with the cell envelope and missing in all sequenced CLas strains [35]. All CLas strains were predicted to be more heavily dependent on additional metabolites, carbohydrates, nucleotides, amino acids and vitamins, and also exhibited marked deficiencies in cell envelope biogenesis, consistent with several lines of published empirical evidence [23, 28, 29, 36].

Of the 95 culturability-related ‘*essential*’ genes of Lcr, 30 encoded hypothetical proteins with unknown function (Additional File 3) and without sequence similarity to any prokaryotic or eukaryotic proteins discoverable by BLASTP in GenBank (Additional File 4). Ten of the 30 hypothetical proteins were predicted to be secreted either via classical [37, 38] or noncanonical [39] secretion pathways and eight proteins were predicted to be membrane localized integral proteins [40] (Additional File 3). The smallest synthetic bacterial genome of *Mycoplasma mycoides* JCVI-syn3.0 (531 kb, 473 protein-coding and 35 RNA genes) contained 84 genes that were involved in the maintenance of cell envelope and 149 genes encoding proteins with unknown biological function [34, 41].

We also analyzed the genomes of 17 uncultured bacterial species, including plant and animal pathogens and insect endosymbionts, for the presence of orthologs of all 95 ‘*essential*’ Lcr genes (Additional File 4). Notably, 49 of the 95 predicted to be required for axenic growth had no orthologs in any of these bacteria, while the remaining 46 had orthologs scattered in one or more of these uncultured bacterial genomes (Table 1). These results support our hypothesis that host-free and autonomous axenic growth of CLas can only be achieved via simultaneous addition of multiple Lcr genes identified as ‘*essential*’, and not by media additives or manipulation of axenic growth conditions alone.

**Table 1:** Frequency of ortholog occurrence of annotated ‘*essential*’ Lcr genes in 13 selected uncultured bacteria

Organism	Occurrence/disease	Genbank/EMBL/DDBJ Acc. #	Genome (bp)	Orthologs^*^ of Lcr genes present
*‘Ca.* Phytoplasma australiense’	Phytopathogen; papaya dieback, grapevine yellows	AM422018	879,959	9/65
Phytoplasma mali	Phytopathogen; apple proliferation disease	CU469464	601,943	8/65
*‘Ca.* Phytoplasma asteris’ OY-M	Phytopathogen; onion yellows phytoplasma	AP006628	853,092	8/65
Maize bushy stunt phytoplasma	Phytopathogen; stunted growth and witches’ broom in maize	NZ_CP015149	576,118	7/65
*‘Ca.* Carsonella ruddii’	Obligate primary endosymbiont of Asian citrus psyllid (*Diaphorina citri*)	AP023214	173,853	5/65
*‘Ca.* Profftella armatura’	Obligate primary endosymbiont of Asian citrus psyllid (*D. citri*)	AP023215	469,264	6/65
*Wolbachia* spp.	Intracellular, secondary endosymbiont of most arthropods and filarial nematodes	NC_021084	1,301,823	24/65
*Wigglesworthia glossinidia*	Obligate primary endosymbiont of tsetse fly (*Glossina morsitans morsitans*)	CP003315	697,724	17/65
*‘Ca.* Baumannia cicadellinicola’	Obligate endosymbiont of glassy-winged (*Homalodisca itripennis*) sharpshooter	CP000238	686,194	21/65
*‘Ca.* Methanoplasma termitum’	Methanogenic; present in higher termite gut (*Cubitermes ugandensis*)	NZ_CP010070	1,488,669	5/65
*Orientia tsutsugamushi*	Obligate intracellular human pathogen; scrub typhus	NZ_LS398548	2,469,803	15/65
*Rickettsia *str. *Iowa*	Obligate intracellular human pathogen; rocky mountain spotted fever	NC_010263	1,268,201	32/65
*‘Ca.* Brocadia sinica’	Environmental; anaerobic ammonia oxidizing	GCA_000949635	4,077,002	24/65

^*^Orthologs for the following 19 ‘*essential*’ *L. crescens* genes are missing across all of the above bacterial genomes: B488_RS00140 (*gtrA*, transmembrane translocation of bactoprenol-linked glucose); B488_RS00510 (*thiS*, thiamine-pyrophosphate biosynthesis); B488_RS00825 (tetratricopeptide repeat containing lipoprotein); B488_RS00940 (DUF4354 family protein); B488_RS01155 (HAD family hydrolase); B488_RS01485 (DUF1344 domain-containing protein); B488_RS03185 (YqcI/YcgG family protein); B488_RS03190 (dTDP-sugar isomerase, biosynthesis of L-rhamnose); B488_RS03535 (ATP synthase subunit B); B488_RS03555 (3-deoxy-7-phosphoheptulonate synthase; aromatic amino acid biosynthesis); B488_RS03865 (dihydroneopterin aldolase, folate biosynthesis); B488_RS04330 (RNase H fold-containing effector); B488_RS05025 (DUF1134 domain-containing protein); B488_RS05105 (GAF domain containing protein); B488_RS05600 (porin family); B488_RS05940 (DUF4164 domain-containing protein); B488_RS06280 (Flp family type IVb pilin); B488_RS06535 (alcohol dehydrogenase); B488_RS06655 (HPr family phosphocarrier protein, energy metabolism regulation)

### Conjugation of culturability-related ‘*essential*’ genes to transient cultures of CLas

Highly efficient, cost-effective and seamless gene synthesis and assembly platforms such as Gibson, Golden Gate and paper-clip assembly etc. [42] and advanced genetic engineering tools [43] have yielded workflows resulting in synthetic, minimal and self-replicative bacterial and yeast genomes [44]. We envisage a similar, albeit less complex and bottom up, approach for conjugal transfer of the 95 culturability-related ‘*essential*’ Lcr genes (under the control of their native promoters) to CLas. Bacterial conjugation is an energy-driven unidirectional DNA transfer process requiring physical contact between donor and recipient cells. Cell-to-cell contact signals the donor bacteria for mating bridge formation and DNA transfer that is largely independent of the size of transferred DNA [45]. Both the transient (co)cultures of CLas [6, 9] or host-free CLas containing mixed biofilms [7, 8] can be potentially used as recipients, as biofilms are known to facilitate conjugation [46].

**Fig. 2:**
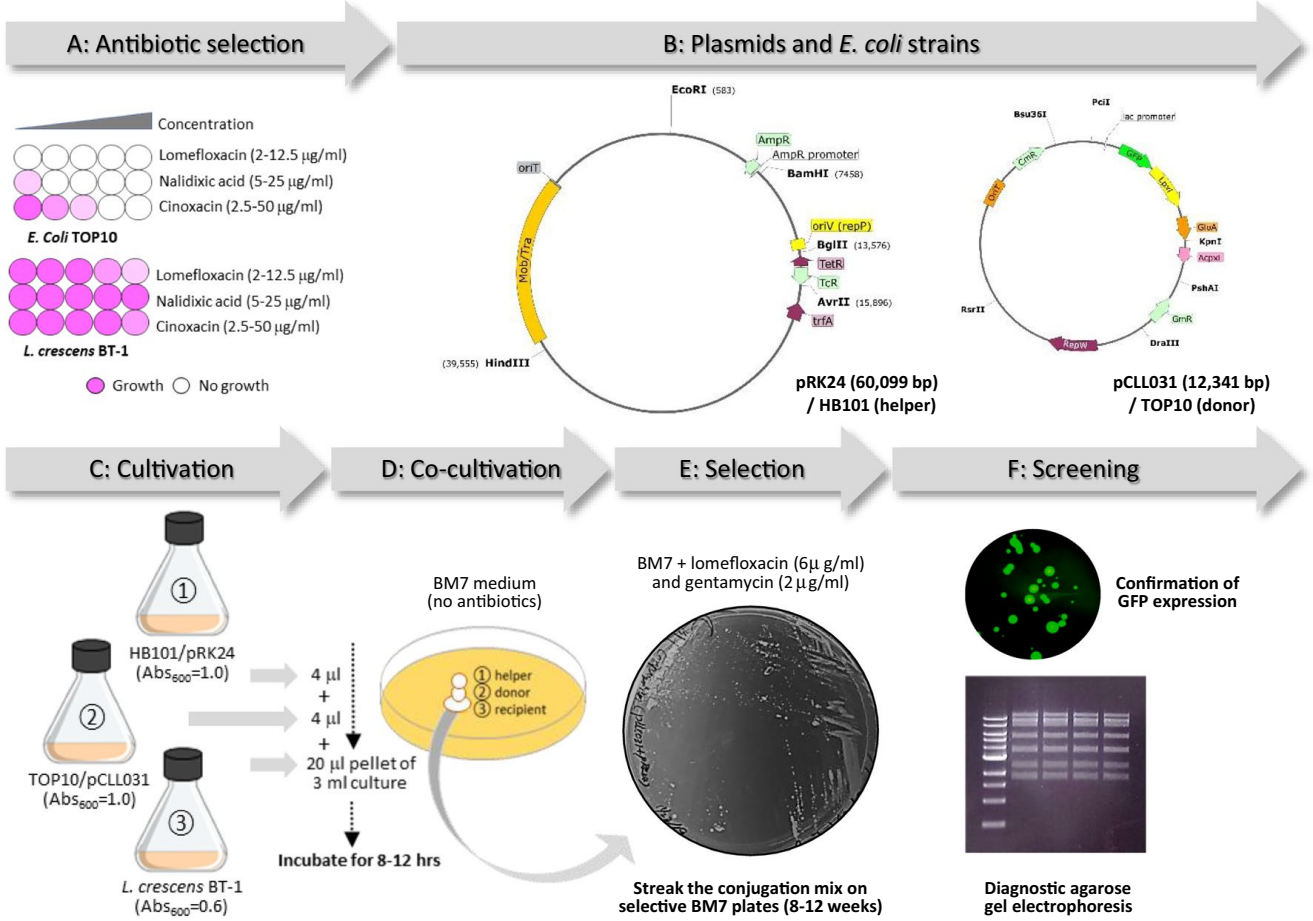
Experimental design for genetic transformation of *Liberibacter crescens* (Lcr) via* E. coli* conjugation. **A** Lomefloxacin effectively suppressed growth of *E. coli* at all the concentrations (2–12.5 μg/ml) tested with no inhibitory effect on Lcr growth (at 2–10 μg/ml). Iodonitrotetrazolium chloride forms a purple formazan dye on reduction indicative of cell growth and viability in a microplate assay. **B** The broad-host range mobilizable plasmid pCLL031 is a derivative of pURF071 (RepW, ColE1, Mob^+^, lacZ, Par^+^, Cm^R^, Gm^R^) containing genes encoding green florescent protein (GFP) driven by a constitutive tryptophan promoter, and glyoxalase A (*gloA*, B488_RS02175), Kdo2-lipid IVA lauroyltransferase (*lpxXL*, B488_RS04675) and acyl carrier protein (*acpXL*, B488_RS04700) with their native promoters. The genotypes of the *E. coli* strains used for conjugation are, HB101 (helper): F- *mcr*B *mrr hsd*S20 (r_B_^−^, m_B_^−^) *rec*A13 *sup*E44 *ara*14 *gal*K2 *lac*Y1 *pro*A2 *rps*L20 (Sm^R^) *xy*15 λ^−^ *leu mtl*1; and TOP10 (donor): F-*mcrA* Δ(*mrr-hsd*RMS*-mcr*BC) Φ80*lac*ZΔM15 Δ*lac*X74 *rec*A1 *ara*D139 Δ(*ara leu*)7697 *gal*U *gal*K *rps*L (Str^R^) *end*A1 *nup*G. **C–D** 4 μl aliquots of overnight cultures (Abs_600_ = 1.0) of helper (HB101) and donor (TOP10) *E. coli* strains were sequentially spotted on top of a 5-day-old 3 ml Lcr culture pellet and cocultured for 8–12 h at 28 °C. **E–F** The coculture mix was streaked on selective BM7 plates (6 μg/ml lomefloxacin and 2 μg/ml gentamycin) for 12 weeks, and the Lcr exoconjugants were verified for the presence of mobilized pCLL031 by restriction digestion analysis and GFP expression

Experimental conditions and counter selection methods for conjugation of large plasmids expressing green florescent protein (GFP) [47] from *E. coli* to Lcr BT-1 were successfully accomplished for the first time and are summarized in Fig. 2. A broad-host range pUFR071 [48] derived *E. coli/*Lcr shuttle plasmid pCLL031 was transferred via conjugation from *E. coli* strain TOP10 (Invitrogen, Waltham, MA) to Lcr, using *E.coli* strain HB101 (Promega, Madison, WI) carrying the conjugative plasmid pRK24 [49] as helper. Phenotypic microarray plate (Biolog Inc., Hayward, CA) assays identified lemoefloxacin (6 μg /ml) as an effective counter selection antibiotic for the recovery of Lcr exoconjugants following mating with *E. coli* on BM7 medium [2]. Gentamicin (2.0 μg/ml) [3] was used for the selection of pCLL031 in Lcr exoconjugants. Simulated growth modeling, accounting for the connectivity of carbon and nitrogen sources, amino acids and vitamins across physiological networks predicted that the Lcr culture medium BM7 was also optimal for in vitro growth of CLas [35]. Alternatively, modified BM7 medium (BM7A) may also be used for recovery of CLas transconjugants. BM7A medium, with increased buffering capacity and reduced medium alkalization, resulted in 1000-fold improved recovery of ‘*viable* and *culturable*’ Lcr cells from 10-day-old cultures [3, 50].

## Conclusions

Currently, 97% of 14,000 cultured bacterial species (across 3500 genera and 38 phyla) belong to just four bacterial phyla (Bacteroidetes, Proteobacteria, Firmicutes and Actinobacteria), but the vast majority remain poorly characterized in vitro [51]. Several bacteria with reduced genomes have remained recalcitrant to axenic growth in vitro, likely because of (a) metabolic deficiencies that cannot be relieved by media supplementation alone, (b) novel regulatory networks that are needed for optimum gene expression and (c) additional genes that required for essential structural, membrane barrier and unknown functions for in vitro growth. Global mutagenesis datasets and modeling of regulatory and metabolic networks in phylogenetically related culturable species can provide valuable insights into gene ‘*essentiality*’ functions and bottom-up implementation of specific synthetic ‘*essentialomes*’ for axenic culturing of economically important pathogens with reduced genomes such as CLas.

## Limitations

Successful axenic culturing of CLas will likely require transfer and expression of a complete set of at least 95 Lcr genes, all of which are simultaneously required. Efforts are underway to obtain a comparative high density transcriptomic roadmap of Lcr and CLas to better understand (a) previously uncharacterized gene regulatory networks and (b) the significance of large numbers of species-specific hypothetical proteins of unknown function present in both the Lcr and CLas genomes.

## Supplementary Information

**Additional file 1: **405 unique ORFs present in the genome of *Liberibacter crescens* but either absent (or truncated) in all sequenced ‘*Ca.* Liberibacter asiaticus’ strains. Genes validated to be essential for growth of *L. crescens* in culture are indicated by asterisk (*).

**Additional file 2: **120 unique ORFs present in the genome of *L. crescens* that are absent in the genomes of all sequenced ‘*Ca.* Liberibacter asiaticus’ strains.

**Additional file 3: **Culturability-related ‘*essential*’ *L. crescens* genes encoding hypothetical proteins that are either secreted or are membrane localized.

**Additional file 4: **Survey of 95 culturability-related ‘*essential*’ genes of *Liberibacter crescens* for homologs present in the genomes of 13 economically/medically important uncultured bacteria. Among the 65 annotated genes, 19 genes had no homologs in any of these bacteria.

## Data Availability

All data generated or analysed during this study are included in this published article and its additional files.

## Abbreviations

CLas: ‘*Candidatus* Liberibacter asiaticus’
Clam: ‘*Ca*. L. americanus’
Lcr: *L. crescens*
